# Effects of *CYP1A2* and *ADORA2A* Genotypes on the Ergogenic Response to Caffeine in Professional Handball Players

**DOI:** 10.3390/genes11080933

**Published:** 2020-08-13

**Authors:** Alejandro Muñoz, Álvaro López-Samanes, Millán Aguilar-Navarro, David Varillas-Delgado, Jesús Rivilla-García, Víctor Moreno-Pérez, Juan Del Coso

**Affiliations:** 1 Exercise and Sport Sciences, Faculty of Health Sciences, Universidad Francisco de Vitoria, 28223 Madrid, Spain; alejandro.munoz@ufv.es (A.M.); millan.aguilar@ufv.es (M.A.-N.); 2 Faculty of Physical Activity and Sports Sciences (INEF), Universidad Politécnica de Madrid (UPM), 28040 Madrid, Spain; jesus.rivilla@upm.es; 3 Exercise Physiology Group, School of Physiotherapy, Faculty of Health Sciences, Universidad Francisco Vitoria, 28223 Madrid, Spain; 4 Faculty of Medicine, Research Unit, Universidad Francisco de Vitoria, 28223 Pozuelo de Alarcon, Madrid, Spain; david.varillas@ufv.es; 5 Sports Research Centre, Miguel Hernandez University of Elche, 03202 Alicante, Spain; vmoreno@goumh.umh.es; 6 Centre for Sport Studies, Rey Juan Carlos University, 28943 Fuenlabrada, Madrid, Spain; juan.delcoso@urjc.es

**Keywords:** ergogenic aids, genetics, polymorphism, team-sport athletes, sport performance

## Abstract

Previous investigations have found that several genes may be associated with the interindividual variability to the ergogenic response to caffeine. The aim of this study is to analyze the influence of the genetic variations in *CYP1A2* (−163C  > A, rs762551; characterized such as “fast” (AA genotype) and “slow” caffeine metabolizers (C-carriers)) and *ADORA2A* (1976T  > C; rs5751876; characterized by “high” (TT genotype) or “low” sensitivity to caffeine (C-carriers)) on the ergogenic response to acute caffeine intake in professional handball players. Thirty-one professional handball players (sixteen men and fifteen women; daily caffeine intake = 60 ± 25 mg·d^−1^) ingested 3 mg·kg^−1^·body mass (bm) of caffeine or placebo 60 min before undergoing a battery of performance tests consisting of a countermovement jump (CMJ), a sprint test, an agility test, an isometric handgrip test, and several ball throws. Afterwards, the handball players performed a simulated handball match (2 × 20 min) while movements were recorded using inertial units. Saliva samples were analyzed to determine the genotype of each player for the −163C  > A polymorphism in the *CYP1A2* gene (rs762551) and for the 1976T  > C polymorphism in the *ADORA2A* gene (rs5751876). In the *CYP1A2*, C-allele carriers (54.8%) were compared to AA homozygotes (45.2%). In the *ADORA2A*, C-allele carriers (80.6%) were compared to TT homozygotes (19.4%). There was only a genotype x treatment interaction for the ball throwing from 7 m (*p* = 0.037) indicating that the ergogenic effect of caffeine on this test was higher in *CYP1A2* AA homozygotes than in C-allele carriers. In the remaining variables, there were no genotype x treatment interactions for *CYP1A2* or for *ADORA2A*. As a whole group, caffeine increased CMJ height, performance in the sprint velocity test, and ball throwing velocity from 9 m (2.8–4.3%, *p* = 0.001–0.022, effect size = 0.17–0.31). Thus, pre-exercise caffeine supplementation at a dose of 3 mg·kg^−1^·bm can be considered as an ergogenic strategy to enhance some neuromuscular aspects of handball performance in professional handball players with low daily caffeine consumption. However, the ergogenic response to acute caffeine intake was not modulated by *CYP1A2* or *ADORA2A* genotypes.

## 1. Introduction

Caffeine (1,3,7-trimethylxanthine) is one of the most commonly used ergogenic aids [1,2], probably because of the ample evidence that supports its effects on sports performance [3,4]. Caffeine was considered a banned substance by several international sport federations and its use was prohibited in competition between 1984 and 2004. However, because of the difficulties to separate between the social use of caffeine from that associated to obtaining ergogenic/performance benefits, this stimulant substance was removed from the list of prohibited substances and moved to the monitoring program of the World Anti-Doping Agency (WADA) to monitor the use of caffeine in sports. The removal of caffeine from the WADA’s prohibited list has provoked an increase in the consumption of caffeine in the last years [2] together with a proliferation of investigations aimed to determine the performance benefits of acute caffeine innate in several sport situations. Different studies have found that ingestion of low-to-moderate doses of caffeine (3–6 mg·kg^−1^·bm) may have the potential to enhance performance in individual [5] and team sports [6]. In addition, previous studies investigating the ergogenic effect of acute caffeine ingestion in team sport disciplines have observed benefits of this supplementation strategy on neuromuscular performance and match-play demands [7,8,9,10]. With this background, caffeine can be considered as an ergogenic substance to increase several aspects of physical performance but a few investigations have suggested that the magnitude of the ergogenic response to acute caffeine intake may vary among individuals [11,12,13]. In these investigations, acute caffeine intake produced an overall ergogenic effect when analyzing all participants as a whole group but researchers detected one or several individuals that did not benefit from caffeine intake (i.e., non-responders to caffeine ergogenicity). Interindividual variations in the ergogenic response to caffeine may be attributed to different factors such as training status [14], sex [15], caffeine ingestion method [16], caffeine dosage [17,18], habitual caffeine intake [19], time-of-day when caffeine is consumed [20,21] and genotype variation [22,23,24]. Other researchers have disputed the existence of non-responders to caffeine as all individuals positively respond to caffeine, to some extent, when using multiple, repeated testing sessions [25].

For these reasons, the influence of genetics on the ergogenic response to caffeine has received more attention in the past few years. Specifically, the interindividual variation in response to caffeine ingestion has been associated to two genes, *CYP1A2* and *ADORA2A* [3]. The cytochrome P450 1A2 is a hepatic enzyme, responsible for ~95% of all caffeine metabolism, which catabolizes caffeine into different dimethylxanthines such as paraxanthine, theophylline, and theobromine [26]. A single nucleotide polymorphism (SNP) within the *CYP1A2* (−163 C > A, rs762551) has been associated with the response to caffeine because it affects the speed of caffeine metabolism [27]. Depending on this SNP in the *CYP1A2*, individuals can be categorized as “fast caffeine metabolizers”(AA genotype) or “slow caffeine metabolizers” (CA/CC genotypes; or C-allele carriers) [28]. With this “metabolic” background, *CYP1A2* AA homozygotes may be individuals with a higher and/or faster response to acute caffeine intake, while the *CYP1A2* C-allele may be associated to the lack of ergogenic response to caffeine. In the last ten years the differences obtained between genotypes in the metabolism of caffeine has attracted the attention of sport science/genetic researchers trying to demonstrate this hypothesis. However, to date, findings have been controversial, with some studies reporting a higher response to caffeine in AA athletes [22,29] and other studies showing a better response to caffeine in C-carrier athletes [30]. Overall, most of the investigations have found that the ergogenic response to caffeine is independent of the *CYP1A2* −163 C > A polymorphism [11,23,24,31,32,33,34,35,36].

*ADORA2A* is another gene that may play a role in the interindividual response to caffeine [28]. *ADORA2A* gene encodes the adenosine receptor A2A; interestingly, it has been found that the main mechanism behind the ergogenic effect of caffeine is its ability to block the fatiguing effects of adenosine [37,38] by acting as an adenosine A1 and A2A receptor antagonist. Thus, variations in the adenosine receptor A2A may affect the capacity of caffeine to effectively block this receptor. In this regard, the 1976T  >  C (rs5751876) SNP in the *ADORA2A* gene has been used to categorize individuals in “high” (TT genotype) or “low” (CC/CT genotype or C-allele carriers) responders to caffeine [24]. The amount of research investigating the effect of this SNP in the ADORA2A is more limited [28], but again, the main outcomes of these investigations are contradictory. One study reported higher improvements in TT athletes compared to C-Carriers in cycling performance [39]. However, the ergogenic response after acute caffeine intake in C-allele carriers has also been found in subsequent investigations [24,40].

To our knowledge, only one previous study developed by Carswell et al. (2020) has investigated the effects of both *CYP1A2* and *ADORA2A* genotypes on the ergogenic response to acute caffeine intake together on exercise performance in endurance athletes [24], but this investigation only used one exercise performance measurement (a 15-min cycling time trial). As the recommendation to assess the response to caffeine in exercise performance is to undertake multiple measurements [25], the aim of the present study is to determine the influence of *CYP1A2* and *ADORA2A* genotypes on response to caffeine during a battery of neuromuscular performance tests and during a simulated game in professional handball players. We hypothesized that acute caffeine ingestion would be ergogenic for improving several aspects of handball performance regardless of participants’ *CYP1A2* and *ADORA2A* genotypes.

## 2. Materials and Methods

### 2.1. Participants

Thirty-one professional handball players participated in this study (age = 23.7 ± 2.8 years, height = 1.78 ± 9.83 m, body mass = 79.2 ± 16.4 kg, handball experience = 12.1 ± 2.6 years) from the same handball club (i.e., First division of the Spanish National League). In this sample, sixteen players were men and fifteen were women. The data on the men and women were merged as the ergogenic response to caffeine is similar in both sexes [41,42]. Ten women participants were tested during the follicular phase of their menstrual cycle and five were tested during the luteal phase according to a mobile application (Mycalendar^®^, Period-tracker, USA). However, recent investigations indicate that the effect of caffeine on exercise performance is similar across all phases of the menstrual cycle [43,44]. All participants were considered as low caffeine consumers (60 ± 25 mg·d^−1^ or 0.76 mg·kg^−1^·d^−1^ ([45])) measured with a valid semi-quantitative caffeine intake questionnaire [46]. We selected low-caffeine consumers to avoid any effect of habituation to caffeine on the results of this investigation. Only professional handball players between 18 and 40 years old were eligible for inclusion. Exclusion criteria were intolerance to caffeine intake, to be suffering from any chronic pathology or an injury in the month prior to the investigation, to be a habitual consumer of caffeine > 100 mg·d^−1^, and to use medicaments or dietary supplements during the study. Participants gave their informed written consent to participate, and the study was approved by the University Ethics Committee (number 22/2019) in accordance with the Declaration of Helsinki.

### 2.2. Experimental Design

A double-blind, placebo-controlled, crossover design was used to assess the ergogenic response to caffeine in the study sample. Each handball player performed two identical experimental trials, separated by a week to allow reliability in the testing, recovery, and substance wash-out. In both trials, the experimental procedures were performed at the same time of the day to avoid the influence of circadian rhythms on performance [20,21]. In each trial, participants ingested an unidentifiable gelatin capsule with either caffeine (3 mg·kg^−1^·bm, Bulk Powders 100% purity, Colchester, UK) or an inert substance (cellulose, Guinama, Valencia, Spain). We selected this dosage of caffeine as acute ingestion of 3 mg·kg^−1^·bm of caffeine has been found to be effective to increase several aspects of players’ physical performance in several team sports [7,8,9,23]). This dosage represents ~4 times the amount of caffeine habitually ingested per day in this sample of players. The capsule containing caffeine or placebo was ingested with 200 mL of water and researchers verified the ingestion. Just 60 min after ingestion (to allow substances absorption [23]), the handball players performed a set of physical tests, as explained below, to determine the ergogenic effects of caffeine on several aspects of handball physical performance. Air temperature (15.3 ± 1.5 °C) and humidity (41 ± 2.6%) were monitored during both trials with a portable weather station (Meteorological Station, Küken, Spain). Verbal encouragement was standardized in all testing to avoid any influence of this aspect on the results of the investigation. Data on the ergogenic effect of caffeine in the subsample of women handball players have been published elsewhere [9].

In the first experimental trial, the handball players provided a saliva sample collected with a sterile buccal swab (300252DNA, Deltalab, Barcelona, Spain), as previously reported [22]. The swabs were placed in polypropylene tubes for protection and sent to the ATG Genetic Studies Center (Madrid, Spain) for analysis in the laboratory. On a later day, the samples were analyzed to determine each participant’s genotype for the −163C  > A polymorphism in the *CYP1A2* gene (rs762551) and for the 1976T  > C polymorphism in the *ADORA2A* gene (rs5751876). The genetic analysis was performed at the end of the physical performance testing, so as not to alter the double-blind randomized experiment. Once the first experiment was concluded, a case-control experimental design was used to determine if the ergogenic effect of caffeine was influenced by variations in the above-mentioned genes. Initially, three groups were created for the *CYP1A2* gene (i.e., CC, CA, and AA) and for the *ADORA2A* gene (i.e., TT, CT, and CC). However, C-allele carriers in the *CYP1A2* gene [24] and C-allele carriers in the *ADORA2A* gene [24,40] were clustered in the same group following previous investigations.

### 2.3. Exercise Protocol

**Figure 1:**
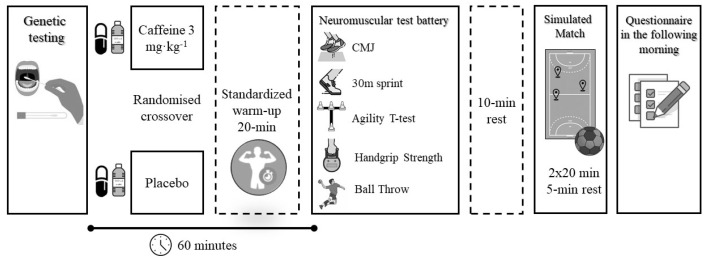
Diagram of the protocol used to assess the influence of polymorphic variants in the *CYP1A2* and *ADORA2A* genes on the ergogenic effect of caffeine on handball-specific performance.

The handball players abstained from any strenuous activity for the 24 h before the experimental trials. Moreover, participants were mandated to refrain from using any source of caffeine for 48 h before the experimental trials. With the aim of standardizing participants’ diets and hydration routines, players were instructed to record the meals and beverages ingested the day before the first experimental trial and to replicate them before the second experimental trial. On the day of testing, the handball players arrived at their habitual training facility at 18.00 h. They then performed a 20-min standardized warm-up that included running and handball-specific exercises such as changes of direction, jumps and ball passes, and throws. Then, participants underwent a battery of neuromuscular tests consisting of a countermovement jump, a sprint velocity test (0–30 m), a modified version of the agility *t*-test, and isometric handgrip strength and a series of different ball throws (7-m and 9-m with and without goalkeeper). One week before the onset of the experiment, a familiarization session that included the execution of all these tests was carried out to avoid the influence of the learning effect on the results of the investigation. After finishing the neuromuscular testing, the players rested for 10 min and then played a simulated handball match (2 × 20 min). At the end of the simulated match, the players were required to fill out a questionnaire about their feelings of muscle power, endurance, and overall perceived exertion during the game, as previously reported [47]. In addition, the morning following the study the participants filled out another questionnaire based on the main side effects associated with caffeine intake during the hours after the trial. A schematic explanation of the experimental protocols is presented in Figure 1.

### 2.4. Countermovement Jump (CMJ)

Participants completed two maximal repetitions of a CMJ (with hands on the hips) and jump height was measured using an infrared beam jump system (Optojump, Microgate, Bolzano, Italy) according to standard methodology [48]. Each participant performed two maximal CMJ interspersed with 45 s of passive recovery. The highest value out of the two jumps was recorded. The test–retest coefficient of variation (CV) was 3.2% [49].

### 2.5. Sprint Test (0–30 m)

Maximal running velocity was measured during a 30-m sprint test. For these measurements, participants had to complete the distance in a straight line as fast as possible while running time was measured by using two photocell gates placed 1.0-m above ground level (Smartspeed, Fusion Sport, Brisbane, Australia). Each sprint was initiated from a standing position, 1-m behind the opening photocell gate, which started a digital timer. Another photocell gate was placed at the finish line to stop the timer. The fastest performance out of two repetitions (separated by a 2-min recovery period) was recorded for subsequent analysis. Test–retest CV was 2.6% [50].

### 2.6. Modified Agility t-Test

Agility was measured using a modified version of the *t*-test, following the protocol outlined by Sassi et al. [51]. Participants began the test with both of their feet behind the starting line. After an acoustic signal, they had to sprint 5-m forward to touch a cone. Then, they shuffled 2.5 m to the left and touched a second cone. After that, they shuffled 5-m to the right and touched a third cone, and then they shuffled 2.50 m back to the left to touch the first cone again and then finally ran backward passing the finish line (which was the same as the starting line). Two electronic time sensors (Smartspeed, Fusion Sport, Brisbane, Australia) were set 1 m above the ground and positioned 3 m apart facing each other on either side of the starting line. Participants began each test 1 m behind the starting line, and the timer started when they passed the first gate. The best performance out of two repetitions (separated by a 2-min recovery period) was recorded for subsequent analysis. Test–retest CV was 1.2% [49].

### 2.7. Isometric Handgrip Strength

Two maximum isometric voluntary contractions were measured in the dominant hand using a calibrated handgrip dynamometer (Takei 5101, Tokyo, Japan). Participant sat with 0 degrees of shoulder flexion, 0 degrees of elbow flexion and the forearm and hand in a neutral position such as previously reported in other studies [52]. The highest value out of two attempts was recorded (separated by a 45-s recovery period). Test–retest CV was 4.1% [52].

### 2.8. Ball Throwing

Players performed a series of ball throws using an official ball with weight and circumference determined by gender. Players were instructed to make two types of throws, one from the penalty line (7 m) and a another from behind the 9 m line with a preparatory three-step run before jumping vertically and throwing the ball. Participants were encouraged to perform each throw toward the center of the goal with maximal velocity [53]. The throws were performed without any opposition and with opposition from the goalkeeper (GK) to simulate different situations [54]. Every participant performed three maximal shots for each type of throw (9 m, 9 m with GK, 7 m, and 7 m with GK). In-between shot recovery was set at 60 s. A sport radar gun (Pocket Radar Ball Coach PR1000-BC, Santa Rosa, CA, USA) recorded ball velocity. The maximum value out of the three shots was recorded. Test–retest CV was 3.5% [52].

### 2.9. Simulated Handball Match-Play

After the handball-specific testing, players participated in a simulated game played on an official handball court. The game consisted of two parts of 20 min with a break of 5 min between them, following the rules of the International Handball Federation (IHF; except for the game duration). To collect time-motion pattern data measurements, all players were equipped with an inertial measurement unit (IMU) with UWB tracking system technology (WIMUPROTM, RealTrack Systems, Almeria, Spain; CV = 2.5–3.5%) [55]. The IMU devices were calibrated and installed around the court as previously described [56]. During these games, player’s substitutions were standardized, and all variables were normalized by playing time. In addition, the number of accelerations, number of decelerations, and impact intensity were recorded.

### 2.10. Side Effects Questionnaire

The morning following the testing, participants were provided with a survey to be filled out about sleep quality, nervousness, gastrointestinal problems, and other discomforts perceived during the hours after the game. This survey included eight items on a yes/no scale and has been previously used to assess side effects derived from caffeine ingestion in the hours following an official or simulated competition [47].

### 2.11. Genetic Testing

DNA isolation was performed on the saliva samples obtained in the first experimental trial, using the NucleoSpin Tissue kit (Macherey-Nagel, Düren, Germany), with minor modifications. Furthermore, DNA concentration and purity were quantified using NanoDrop one (Thermo Fisher Scientific, Wilmington, DE, USA). The samples analyzed were for the *CYP1A2* (rs762551) and *ADORA2A* (rs5751876) and single nucleotide polymorphisms. Genotyping was successful (i.e., successful determinations for both polymorphisms) in all participants.

### 2.12. Statistical Analysis

The results obtained in the placebo and caffeine trials are presented as means ± standard deviation for the whole group of participants and for each genotype. As a whole group, the differences between treatments (i.e., caffeine vs. placebo) were determined using paired t tests. A two-way analysis of variance (ANOVA) with one between-group factor (genotype) and one within-subject factor (treatment) was conducted for each gene to determine the influence of *CYP1A2* and *ADORA2A* genotypes on the ergogenic response to caffeine. After a significant F test in the genotype * treatment interaction, LDS post-hoc analysis was used in each caffeine-placebo pairwise comparison within each genotype to determine differences in the ergogenic response to caffeine. Chi-square tests were used to determine differences in the prevalence of side effects between the different genotypes of the *CYP1A2* and *ADORA2A* genes. Effect size (ES) was also calculated in all pairwise comparisons, using Cohen’s d ± 95% confidence intervals (CI), to assess the magnitude of the ergogenic response to caffeine in each phenotype under investigation. ES was interpreted according to the following ranges: <0.2, trivial; 0.2–0.6, small; 0.6–1.2, moderate; 1.2–2.0, large; 2.0–4.0, very large; and >4.0, extremely large [57]. The Hardy-Weinberg equilibrium (HWE) was tested for each polymorphism using *χ*^2^ tests. All the analyses were performed with the statistical package SPSS version 20.0 (SPSS Inc., Chicago, IL, USA).

## 3. Results

Table 1 presents the number and frequency of participants within each genotype for *CYP1A2* and *ADORA2A*. The sample distribution for the −163C  > A polymorphism of the *CYP1A2* gene (*p* = 0.315) and for the 1976T  > C polymorphism of the *ADORA2A* gene (*p* = 0.781) met the HWE.

### 3.1. CYP1A2

As a whole group, and in comparison to the placebo trial, acute caffeine intake increased CMJ height (32.29 ± 7.43 vs. 33.55 ± 7.58 cm; *p* = 0.001, ES = 0.17 [0.08, 0.26]), reduced the time to complete the sprint velocity test (4.73 ± 0.40 vs. 4.61 ± 0.42 s; *p* = 0.022, ES = −0.29 [−0.38, −0.20]), and enhanced the ball velocity in the throwing from 9 m (82.55 ± 7.64 vs. 84.90 ± 7.32 km/h; *p* = 0.008, ES = 0.31 [0.22, 0.40]). However, caffeine intake did not modify the time to complete the MATT (5.83 ± 0.55 and 5.81 ± 0.45 s; *p* = 0.686, ES = −0.04 [−0.13, 0.05]), the strength in the IHS test (45.18 ± 12.21 and 46.45 ± 11.45 kg; *p* = 0.054, ES = 0.11 [0.00, 0.20]), nor the ball velocity in the BT7M (82.69 ± 9.25 and 84.03 ± 8.91 km/h; *p* = 0.065, ES = 0.15 [−0.06, 0.24]), BT7M + GK (81.97 ± 7.72 and 82.62 ± 7.38 km/h; *p* = 0.492; ES = 0.09 [−0.10 0.18]), and BT9M + GK throw tests (83.72 ± 7.40 and 85.59 ± 7.10; *p* = 0.093, ES = 0.26 [−0.17, 0.35]). Table 2 presents data on all these neuromuscular performance tests with the ingestion of caffeine or a placebo depending on the *CYP1A2* genotype. There was only a genotype x treatment interaction for the ball throws from 7 m (*p* = 0.037). In this performance test, the post-hoc analysis revealed that only *CYP1A2* AA homozygotes reported an ergogenic effect of caffeine (*p* = 0.013) while C-allele carriers did not improve ball velocity from 7 m (*p* = 0.932). No other genotype * treatment interactions were found in the remaining tests.

As a whole group, and in comparison to the placebo trial, acute caffeine intake did not modify the frequency of ACC (18.75 ± 1.57 and 19.13 ± 1.16 number/min; *p* = 0.178, ES = 0.28 [−0.19, 0.37]), DEC (19.00 ± 1.37 vs. 18.72 ± 1.42 number/min; *p* = 0.051, ES = −0.20 [−0.29, 0.11]) nor the frequency of BI (23.87 ± 11.46 and 26.25 ± 13.57 number/min; *p* = 0.556, ES = 0.19 [−0.10, 0.28]) during the simulated match. Table 3 depicts match movement patterns with caffeine and placebo depending on the *CYP1A2* genotype. There was not any genotype * treatment interaction in these variables.

### 3.2. ADORA2A

Table 4 presents data on all the neuromuscular performance tests with the ingestion of caffeine or a placebo depending on the *ADORA2A* genotype. There was not any genotype * treatment interaction in any of the neuromuscular tests.

Table 5 depicts match movement patterns with caffeine and placebo depending on the *ADORA2A* genotype. There was not any genotype x treatment interaction in the variables obtained during the simulated match.

### 3.3. Prevalence of Side Effects

Table 6 depicts the frequency of caffeine-associated side effects with caffeine and placebo in professional handball players with different genotypes in the *CYP1A2* and *ADORA2A* genes. Overall, the prevalence of side effects with caffeine ingestion was similar in the genotype groups of *CYP1A2* and *ADORA2*. However, C-allele carriers in the *CYP1A2* gene presented a higher rating of insomnia than AA homozygotes (*p* = 0.023). In addition, TT homozygotes in the *ADORA2A* gene presented a higher frequency of increased urine production (*p* < 0.001) and increased activeness (*p* = 0.016).

## 4. Discussion

The aim of this study was to analyze the influence of genetic variations in the *CYP1A2* and *ADORA2* genes on the ergogenic response to acute caffeine intake in professional handball players. The main finding of the present study is that caffeine ingestion enhanced CMJ height, performance in the sprint velocity test, and ball throwing velocity from 9 m suggesting an ergogenic effect of acute caffeine ingestion for handball physical performance. However, despite the high number of performance tests included in this investigation, and the analysis of two genetic polymorphisms previously associated to interindividual variations in the response to acute caffeine intake, there was only a genotype x treatment interaction for the ball throwing from 7 m (*p* = 0.037) indicating that the ergogenic effect of caffeine on this test was higher in *CYP1A2* AA homozygotes than in C-allele carriers. Overall, these outcomes indicate that caffeine has the ability to enhance handball-specific performance while *CYP1A2* and *ADORA2A* polymorphisms minimally altered the ergogenic response to caffeine. As a result, it seems safe to suggest that pre-exercise caffeine supplementation at a dose of 3 mg·kg^−1^ bm can be considered as an ergogenic strategy to enhance some neuromuscular aspects of handball performance. Furthermore, the magnitude of the ergogenic effect of caffeine was, overall, similar in players with different genotypes in *CYP1A2* (−163C  > A; rs762551) and *ADORA2A* (1976T  > C; rs5751876) genes. Although caffeine was considered a banned substance in sports and its use was prohibited in competition between 1984 and 2004 (an adverse analytical finding was reported when urinary caffeine concentration surpassed 12 μg/mL [1]), the wide accessibility and popularity of caffeine-containing sources, and the relatively low prevalence of side effects reported when caffeine is ingested acutely in moderate doses suggest that the use of caffeine supplementation before sports competition cannot be considered as a unethical behavior in sports. The current investigation reinforces this idea because it suggests that high-performance athletes can benefit from acute ingestion irrespective of their *CYP1A2* and *ADORA2A* genotype.

### 4.1. CYP1A2

In the current investigation, acute caffeine intake (3 mg·kg^−1^·bm) was effective to increase jump height in a countermovement jump (+3.9%). This ergogenic effect of caffeine is a recurrent finding in the literature as several investigations have found an effect of similar magnitude in basketball players [11], volleyball players [49], swimmers [13], and resistance-trained individuals [31]. Acute caffeine intake was also ergogenic to enhance sprint performance as it reduced the time to complete a 30-m sprint test (−2.6%). These benefits were present without any *CYP1A2* genotype x treatment interaction suggesting that players with the AA genotype and those carrying the C-allele may benefit from acute caffeine intake to increase muscle power and sprint-related activities. Interestingly, caffeine did not produce any effect on the time employed to complete the modified version of the *t*-test, independently of the *CYP1A2* genotype of the individuals. As this is a recurrent finding in the literature [11], it may suggest that the effect of caffeine to increase running performance is not present in agility tasks that require changes of direction. It is worth mentioning that no previous study has analyzed the effect of caffeine and the interaction with the *CYP1A2* genotype in handball. In the current investigation, acute caffeine intake enhanced the ball velocity in the throwing from 9 m while other non-statistically significant benefits were found in other handball-specific tests and in a simulated match when comparing data of the placebo and caffeine trials as a whole group. In these handball-specific testing, only a *CYP1A2* genotype x treatment interaction was found in the BT7M test (Table 2). These data are in agreement with the results from other intermittent sports such as tennis in which caffeine ingestion improved the number of successful shots during a serve tennis performance test with minimal influence of the *CYP1A2* genotype [36]. These combined results, in terms of genotype, reinforce the idea that the *CYP1A2* genotype has little influence on the ergogenic response to caffeine, as previously found in other forms of exercise [11,23,24,32,33,34,35,36]. Thus, despite findings associated with a higher ergogenic response to caffeine in *CYP1A2* AA athletes with respect to C-allele athletes [22,29], the current investigation suggests that the ergogenic effect of caffeine on neuromuscular performance may be independent of the −163 C > A *CYP1A2* genotype, at least in handball players. In this regard, caffeine may be recommended for both *CYP1A2* AA and C-allele carriers seeking to enhance their physical performance with caffeine in a safe manner as the prevalence of side effects was comparable in both groups (Table 6). Of note, C-allele carriers in the *CYP1A2* gene showed higher ratings of insomnia, and thus should evaluate the use of caffeine to increase their performance in evening training sessions as they may experience a higher frequency of insomnia when using this supplementation strategy. These findings do not dispute the notion that the AA genotype increases the metabolism of caffeine, as previously found [28]. However, it is possible that fast/slower caffeine metabolism does not produce any benefit in terms of response to acute caffeine intake as the subproducts of caffeine metabolism may also be ergogenic [58,59]. This latter speculation warrants further investigation.

### 4.2. ADORA2A

Our study showed no influence of the *ADORA2A* genetic variation (TT vs. C-allele carriers) on the ergogenic response to caffeine. The literature on this topic is scarce in comparison to the *CYP1A2* gene, but again, the current finding is supported by the existing studies. Loy et al. [39] found that only *ADORA2A* TT homozygotes benefited from caffeine ingestion while C-allele carriers in this gene did not obtain any ergogenic benefit during a 10 min all-out time trial. This pioneer investigation suggested that C-allele carriers may be considered as non-responders to caffeine, while more recent evidence disputed this notion. Grgic et al. [40] have found that *ADORA2A* C-allele carriers benefited from caffeine in terms of exercise performance in a battery of neuromuscular tests, although their response could not be compared to TT homozygotes as none of their 22 participants presented this genotype. Carswell et al. [24] have found that both TT and C-allele carriers enhanced exercise performance by a similar degree during a 15-min cycling time trial. Interestingly, 83.3% of the players with the *ADORA2A* TT genotype in the current investigation reported increased urine production in the hours after caffeine ingestion, a percentage higher than in C-allele carriers (Table 6). In addition, players with the *ADORA2A* TT genotype also reported a higher rating of increased activeness than C-allele counterparts, suggesting that TT may be more prone to suffer some of the most common caffeine-induced drawbacks when using acute caffeine intake. In this regard, TT homozygotes should value the use of caffeine in terms of benefits/drawbacks, test its effectiveness during training or simulated competition and discontinue supplementation in the case of continued side-effects.

### 4.3. Strengths and Limitations

To our knowledge, this is the first study to analyze the influence of two genotypes (*ADORA2A* and *CYP1A2A*) on the ergogenic response to caffeine by using multiple exercise performance testing. However, some limitations are present such as: (a) the limited sample size of handball players; (b) the reduced number of TT homozygotes in the *ADORA2A* gene; (c) the lack of blood biomarkers that could have helped to provide an explanation of the main mechanisms behind our findings; (d) the use of only one dose of caffeine; (e) the use of individuals with low habituation to caffeine. Future studies are required to understand the possible role of other polymorphism in *CYP1A2* and *ADORA2A*, in addition to investigating the influence of other genes, to completely unveil the influence of genetics on the ergogenic response to caffeine. The investigation of higher acute doses of caffeine and the study of the genetics-caffeine’s response interaction in individuals habituated to caffeine may be pertinent in future investigations as the ergogenic response to caffeine may be influenced by genetics with higher acute/chronic doses of caffeine.

## 5. Conclusions

In the light of the current outcomes, caffeine supplementation can be considered as an ergogenic strategy to enhance some neuromuscular aspects of handball performance in players with low habituation to caffeine. However, the magnitude of the ergogenic effect of caffeine was, overall, similar in players with different genotypes in *CYP1A2* (−163C  > A; rs762551) and *ADORA2A* (1976T  > C; rs5751876) genes. The only exception is that only AA *CYP1A2* players obtained higher benefits with caffeine on one type of handball-specific throw performance with respect to C-allele carriers. Thus, caffeine may be used to enhance physical performance in professional handball players, irrespective or their *CYP1A2* and *ADORA2A* genotypes. Of note, as there is a progressive tolerance to the ergogenic benefit of caffeine with chronic ingestion [60], together with a progressive increase in the prevalence of side effects [61] professional handball players should avoid using caffeine on a daily basis to prevent habituation. The use of caffeine to increase performance in team-sports should be evaluated compared to its side-effects [62], especially in *CYP1A2* C-allele carriers and *ADORA2A* TT homozygotes as insomnia, diuresis, and excessive activeness may be reported by athletes with these genotypes.

## Figures and Tables

**Table 1:** Number and genotype frequency of the variations in the −163C  >  A polymorphism of the *CYP1A2* gene and in the 1976T  >  C polymorphism of the *ADORA2A* gene in a sample of thirty-one professional handball players.

*CYP1A2*				*ADORA2A*			
Genotype	Number (Frequency)			Genotype	Number (Frequency)		
AA	14 (45.2)	AA	14 (45.2)	TT	6 (19.4)	TT	6 (19.4)
CA	15 (48.4)	C-allele	17 (54.8)	CT	16 (51.6)	C-allele	25 (80.6)
CC	2 (6.4)			CC	9 (29.0)		

**Table 2:** Performance variables during neuromuscular tests with ingestion of 3 mg·kg^−1^ bm of caffeine or a placebo in professional handball players with different genotypes in the −163C  > A polymorphism of the *CYP1A2* gene.

Variable (Units)	*CYP1A2* Genotype	Placebo	Caffeine	% Change	ES [95%CI]	Interaction
**CMJ (cm)**	AA	32.91 ± 3.52	34.02 ± 4.44	3.4	0.28 [0.08, 0.48]	0.903
	C-allele	31.88 ± 9.22	33.25 ± 9.18	4.3	0.15 [0.01, 0.31]	
**SV (s)**	AA	4.75 ± 0.41	4.46 ± 0.27	−5.0	−0.84 [−1.04, −0.63]	0.140
	C-allele	4.77 ± 0.43	4.70 ± 0.49	−1.5	−0.15 [−0.32, −0.01]	
**MATT (s)**	AA	5.69 ± 0.38	5.70 ± 0.24	0.1	0.03 [−0.17, 0.23]	0.451
	C-allele	5.92 ± 0.65	5.89 ± 0.55	−0.5	−0.05 [−0.21, 0.11]	
**IHS (kg)**	AA	48.21 ± 14.51	48.24 ± 13.14	0.1	0.00 [−0.20, 0.20]	0.069
	C-allele	42.52 ± 9.46	44.88 ± 11.03	5.5	0.23 [0.07, 0.39]	
**BT7M (km/h)**	AA	83.62 ± 9.66	86.85 ± 9.49	3.9	0.34 [0.14, 0.54]	0.037 *
	C-allele	81.94 ± 9.15	81.75 ± 7.99	−0.2	−0.02 [−0.19, 0.14]	
**BT7M + GK (km/h)**	AA	82.15 ± 8.53	85.46 ± 8.24	4.0	0.39 [0.19, 0.59]	0.061
	C-allele	81.81 ± 7.27	80.31 ± 5.90	−1.8	−0.23 [−0.39, −0.06]	
**BT9M (km/h)**	AA	82.31 ± 8.79	85.62 ± 7.52	4.0	0.40 [0.20, 0.60]	0.207
	C-allele	82.75 ± 6.87	84.31 ± 7.35	1.9	0.22 [0.06, 0.38]	
**BT9M + GK (km/h)**	AA	82.77 ± 8.11	86.46 ± 7.49	4.5	0.47 [0.27, 0.67]	0.147
	C-allele	84.50 ± 6.95	84.88 ± 6.93	0.4	0.05 [−0.11, 0.22]	

Abbreviations: CMJ: countermovement jump; SV: sprint velocity test; MATT; modified agility *t*-test; IHS: isometric handgrip strength; BT7M: ball throw 7-m, BT7M + GK: ball throw 7-m with goalkeeper; BT9M: ball throw 9-m; BT9 + GK: ball throw 9-m with goalkeeper; ES: effect size; CI: confidence interval. * Statistical significance was set at *p* < 0.05.

**Table 3:** Match-play movement patterns with the ingestion of 3 mg·kg^−1^ bm of caffeine or a placebo in professional handball players with different genotypes in the −163C  > A polymorphism of the *CYP1A2* gene.

Variable (Units)	*CYP1A2* Genotype	Placebo	Caffeine	% Change	ES [95%CI]	Interaction
**ACC (number/min)**	AA	18.89 ± 1.28	18.79 ± 0.94	−0.5	−0.09 [−0.29, 0.11]	0.090
	C-allele	18.64 ± 1.64	19.40 ± 1.28	4.1	0.52 [0.35, 0.68]	
**DEC (number/min)**	AA	19.09 ± 1.13	18.46 ± 1.07	−3.3	−0.57 [−0.77, −0.37]	0.344
	C-allele	18.92 ± 1.58	18.93 ± 1.66	0.1	0.01 [−0.16, 0.17]	
**BI (number/min)**	AA	20.57 ± 13.37	21.64 ± 13.65	5.2	0.08 [−0.12, 0.28]	0.307
	C-allele	26.58 ± 9.14	30.05 ± 12.65	13.1	0.31 [0.15, 0.48]	

Abbreviations: ACC: acceleration; DEC: decelerations; BI: body impacts; ES: effect size; CI: confidence interval.

**Table 4:** Performance variables during neuromuscular tests with ingestion of 3 mg·kg^−1^ bm caffeine or a placebo in professional handball players with different genotypes in the 1976T  > C polymorphism of the *ADORA2A* gene.

Variable (Units)	*ADORA2A* Genotype	Placebo	Caffeine	% Change	ES [95%CI]	Interaction
**CMJ (cm)**	TT	32.70 ± 8.8	33.45 ± 8.5	2.3	0.09 [−0.38, 0.55]	0.602
	C-allele	33.10 ± 7.7	34.24 ± 7.7	3.4	0.15 [0.04, 0.26]	
**SV (s)**	TT	4.75 ± 0.44	4.68 ± 0.45	1.4	−0.16 [−0.62, 0.31]	0.866
	C-allele	4.73 ± 0.40	4.59 ± 0.42	3.0	−0.34 [−0.45, −0.23]	
**MATT (s)**	TT	6.05 ± 0.65	6.06 ± 0.59	−0.1	0.02 [−0.45, 0.48]	0.600
	C-allele	5.76 ± 0.52	5.74 ± 0.39	0.3	−0.04 [−0.15, 0.07]	
**IHS (kg)**	TT	40.67 ± 10.17	43.70 ± 12.38	7.5	0.27 [−0.20, 0.73]	0.575
	C-allele	46.86 ± 12.65	47.30 ± 11.81	0.9	0.04 [−0.07, 0.15]	
**BT7M (km/h)**	TT	79.20 ± 9.47	77.80 ± 3.63	−1.8	−0.20 [−0.66, 0.27]	0.879
	C-allele	83.88 ± 9.34	85.20 ± 9.01	1.6	0.14 [0.03, 0.25]	
**BT7M + GK (km/h)**	TT	78.80 ± 6.91	77.40 ± 1.52	−1.8	−0.28 [−0.74, 0.18]	0.151
	C-allele	82.88 ± 7.79	83.44 ± 7.63	0.7	0.07 [−0.04, 0.18]	
**BT9M (km/h)**	TT	79.60 ± 4.22	79.80 ± 4.66	0.3	0.04 [−0.42, 0.51]	0.255
	C-allele	83.32 ± 7.97	85.88 ± 7.25	3.1	0.34 [0.22, 0.45]	
**BT9M + GK (km/h)**	TT	78.40 ± 3.65	80.40 ± 4.16	2.6	0.51 [0.04, 0.98]	0.443
	C-allele	84.80 ± 7.39	86.48 ± 7.07	2.0	0.23 [0.12, 0.34]	

Abbreviations: CMJ: countermovement jump; SV: sprint velocity test; MATT; modified agility *t*-test; IHS: isometric handgrip strength; BT7M: ball throw 7-m, BT7M + GK: ball throw 7-m with goalkeeper; BT9M: ball throw 9-m; BT9 + GK: ball throw 9-m with goalkeeper; ES: effect size; CI: confidence interval.

**Table 5:** Match-play movement patterns with the ingestion of 3 mg·kg^−1^ bm of caffeine or a placebo in professional handball players with different genotypes in the 1976T  >  C polymorphism of the *ADORA2A* gene.

Variable (Units)	*ADORA2A* Genotype	Placebo	Caffeine	% Change	ES [95%CI]	Interaction
**ACC (number/min)**	TT	18.25 ± 2.97	19.91 ± 1.81	9.1	0.67 [0.20, 1.15]	0.409
	C-allele	18.87 ± 1.07	18.94 ± 0.90	0.4	0.07 [−0.04, 0.18]	
**DEC (number/min)**	TT	18.97 ± 2.60	18.96 ± 2.69	−0.1	0.00 [−0.47, 0.46]	0.810
	C-allele	19.00 ± 0.98	18.66 ± 1.00	−1.8	−0.34 [−0.46, −0.23]	
**BI (number/min)**	TT	24.93 ± 11.62	30.16 ± 16.08	21.0	0.37 [−0.09, 0.84]	0.753
	C-allele	23.61 ± 11.65	25.31 ± 13.10	7.2	0.14 [0.03, 0.25]	

Abbreviations: ACC: acceleration; DEC: decelerations; BI: body impacts. ES: effect size; CI: confidence interval.

**Table 6:** Prevalence of side effects with the ingestion of 3 mg·kg^−1^ bm of caffeine or a placebo in professional handball players with different genotypes in the −163C  > A polymorphism of the *CYP1A2* gene and in the 1976T  > C polymorphism of the *ADORA2A* gene.

*YP1A2* Genotype				*ADORA2A* Genotype			
**Variable (Frequency)**	**Placebo**	**Caffeine**	***p***	**Variable**	**Placebo**	**Caffeine**	***p***
**Insomnia**	**22.6**	**48.4**					
AA	21.4	28.6	0.023 *	TT	16.7	33.3	0.174
C-allele	23.5	64.7		C-allele	20.0	56.0	
**Increased urine production**	**25.8**	**45.2**					
AA	14.3	35.7	0.732	TT	16.7	83.3	<0.001 *
C-allele	35.3	52.9		C-allele	28.0	36.0	
**Gastrointestinal problems**	**9.7**	**29.2**					
AA	14.3	35.7	0.193	TT	0	33.3	0.218
C-allele	5.9	11.8		C-allele	12.0	20.0	
**Increased activeness**	**29.2**	**16.1**					
AA	14.3	14.3	0.739	TT	0	50.0	0.016 *
C-allele	35.6	19.8		C-allele	36.1	8.2	
**Headache**	**16.1**	**25.8**					
AA	7.1	28.6	0.363	TT	33.3	16.7	0.108
C-allele	23.5	23.5		C-allele	12.0	28.0	
**Irritability**	**29.2**	**29.2**					
AA	28.6	21.4	0.636	TT	16.7	33.3	0.558
C-allele	17.6	23.5		C-allele	24.0	28.0	
**Muscular pain**	**29.0**	**35.5**					
AA	14.3	35.7	0.251	TT	66.7	50.0	0.094
C-allele	41.2	35.3		C-allele	20.0	32.0	
**Tachycardia/palpitations**	**12.9**	**35.5**					
AA	14.3	42.9	0.632	TT	33.3	33.3	0.282
C-allele	11.8	29.4		C-allele	12.0	36.0	

The comparison for the placebo-caffeine in the whole group of participants is in bold. * Statistical significance was set at *p* < 0.050.
